# Does Global Progress on Sanitation Really Lag behind Water? An Analysis of Global Progress on Community- and Household-Level Access to Safe Water and Sanitation

**DOI:** 10.1371/journal.pone.0114699

**Published:** 2014-12-11

**Authors:** Oliver Cumming, Mark Elliott, Alycia Overbo, Jamie Bartram

**Affiliations:** 1 Department of Disease Control, Faculty of Infectious Tropical Disease, London School of Hygiene and Tropical Medicine, London, United Kingdom; 2 Department of Civil, Construction and Environmental Engineering, University of Alabama, Tuscaloosa, Alabama, United States of America; 3 Water Institute, Gillings School of Global Public Health, University of North Carolina at Chapel Hill, Chapel Hill, North Carolina, United States of America; California Department of Public Health, United States of America

## Abstract

Safe drinking water and sanitation are important determinants of human health and wellbeing and have recently been declared human rights by the international community. Increased access to both were included in the Millennium Development Goals under a single dedicated target for 2015. This target was reached in 2010 for water but sanitation will fall short; however, there is an important difference in the benchmarks used for assessing global access. For drinking water the benchmark is community-level access whilst for sanitation it is household-level access, so a pit latrine shared between households does not count toward the Millennium Development Goal (MDG) target. We estimated global progress for water and sanitation under two scenarios: with equivalent household- and community-level benchmarks. Our results demonstrate that the “sanitation deficit” is apparent only when household-level sanitation access is contrasted with community-level water access. When equivalent benchmarks are used for water and sanitation, the global deficit is as great for water as it is for sanitation, and sanitation progress in the MDG-period (1990–2015) outstrips that in water. As both drinking water and sanitation access yield greater benefits at the household-level than at the community-level, we conclude that any post–2015 goals should consider a household-level benchmark for both.

## Introduction

In 2012, the United Nations (UN) Secretary General declared that the water component of MDG target 7c, to reduce by half the proportion of people without access to safe drinking water, had been met, five years ahead of the 2015 deadline [1]. By contrast, the sanitation target, to reduce by half the proportion of people without access to safe sanitation, was declared seriously off-track and unlikely to be achieved [2]. Whereas 780 million people are estimated to lack access to an ‘improved’ source of drinking water, an estimated 2.5 billion lack access to an ‘improved’ sanitation facility [2]. This apparent deficit, with global progress on extending access to safe sanitation lagging behind that of water, has been coined the ‘sanitation crisis’ and has contributed to various calls to action being issued [3].

The current MDG target for water and sanitation and the methods for monitoring its progress are part of a longer-term evolution of international goal setting and monitoring for water and sanitation [4]. Although there is one joint target for water and sanitation – “to halve, by the year 2015, the proportion of people without sustainable access to safe drinking water and basic sanitation” [2] – the respective benchmarks for water and sanitation differ significantly with regard to the level of access deemed adequate. For water, the benchmark for ‘improved’ is community-level access and, for sanitation, it is household-level access [2]. This decision to establish different benchmarks was seemingly pragmatic; a combination of evidence and experience but also realism in terms of what could be achieved at that time in light of existing levels of progress, resource constraints, and historic levels of ambition. In addition, keeping the water target at the level of the community may have been considered a progressive measure to incentivize reaching those with distant and unsafe water, rather than improving the level of access of those already served at the community-level through subsidized household connections.

The inclusion of water and sanitation within the MDG framework reflects the important contribution of these basic services to human health and wellbeing as well as to the realization of human rights. It has been estimated that as much as 6.6% of the global burden of disease is attributable to poor water, sanitation and hygiene, and this problem is heavily concentrated in low income settings [5]. In particular, diarrhoeal diseases, largely preventable with safe water, sanitation and hygiene, persist as a leading cause of child deaths globally [6]. Water and sanitation also contribute to economic development [7], education [8] and improving the nutritional status of children [9]. Beyond these specific benefits, access to “sufficient, safe, accessible and affordable” water and sanitation is recognized as a human right [10].

The extent to which these benefits differ between community- and household-level access to water and sanitation is unclear but that they do differ significantly is well established and generally accepted [11] and this is discussed further below. The human rights considerations of sufficiency, safety, accessibility and affordability are also inextricably linked to where services are located in relation to the household. The benchmarks for water and sanitation established under the MDG target however were not consistent in this regard; the minimum requirement for water was a protected community-level source, such as a tubewell, but for sanitation it was a household-level sanitation facility, such as a household pit latrine. It is striking that within one MDG target, such different benchmarks should have been established in relation to both human rights obligations and the potential health benefits.

In the coming year, a new set of development goals will be agreed and an ‘illustrative’ proposal for these has been put forward which includes a dedicated universal water and sanitation access goal [12]. However the meaning of this goal is contingent on which benchmarks will be used in monitoring changes in access to water and sanitation. In this analysis, we assess progress on water and sanitation access from 1990 through 2015 under two scenarios: the first with a community-level benchmark for both water and sanitation; and the second with a household-level benchmark for both. We refashion the current MDG target definitions of ‘improved’ in order to critically assess progress in bringing water and sanitation closer to the household where health and other benefits are greatest. A better understanding of trends in global progress for community- and household-level access to these services can support current debates as to the appropriate level of ambition and focus for new international goals on water and sanitation.

## Methods

This study uses the same data sources used by the World Health Organization (WHO)/United Nations Children's Fund (Unicef) Joint Monitoring Programme (JMP) to assess national, regional and global progress toward the water and sanitation MDG target. This analysis models global progress on water and sanitation under the two scenarios described in Table 1; the first with a community-level benchmark for access and the second with a household-level benchmark. Under the ‘community-level benchmark’ scenario, *both* community-level access (i.e. a public water point or sanitation facilities that are shared by more than one household) *and* household-level access are classified as improved. Under the ‘household-level benchmark’ scenario *only* household-level access for both water and sanitation (i.e. only individual household water sources and sanitation facilities) are classified as improved.

**Table 1:** Description of scenarios and benchmark definitions.

Scenario	Benchmark	Change from MDG target definition	Water technology categories included	Sanitation technology categories included
0.	**“Improved” as currently defined by the WHO/Unicef Joint Monitoring Programme**	N/A	Piped water into dwelling, yard, or plot; Public tap or standpipe; Tubewell or borehole; Protected dug well; Protected spring; Rainwater	Flush or pour-flush toilet to piped sewer system, septic tank, or pit latrine; Ventilated improved pit latrine (VIP); Pit latrine with slab; Composting toilet (if it is used by a single household)
1.	**Community-level water and sanitation (includes access at any point within the community, including the household)**	Includes shared ‘improved’ sanitation facilities	Piped water into dwelling, yard, or plot; Public tap or standpipe; Tubewell or borehole; Protected dug well; Protected spring; Rainwater	Flush or pour-flush toilet to piped sewer system, septic tank, or pit latrine; Ventilated improved pit latrine (VIP); Pit latrine with slab; Composting toilet; Shared flush or pour-flush toilet to piped sewer system, septic tank, or pit latrine; Shared VIP latrine; Shared pit latrine with slab (whether used by one household or shared by multiple households)
2.	**Household-level water and sanitation**	Excludes shared ‘improved’ water sources	Piped water into dwelling, yard, or plot; Tubewell or borehole in dwelling, yard, or on-plot; Dug well in dwelling, yard, or on-plot; Rainwater	Flush or pour-flush toilet to piped sewer system, septic tank, or pit latrine; Ventilated improved pit latrine (VIP); Pit latrine with slab; Composting toilet (only if it is used by a single household and not shared by multiple households)

It is necessary to ‘gap fill’ for certain countries and years where data are unavailable and for this we use an alternative approach to that currently used by the JMP. The methods are described in three parts: (1) the approach to gap-filling for missing data; (2) estimation of progress under Scenario 1 with a community-level access benchmark; (3) estimation of progress under Scenario 2 with a household-level access benchmark.

In total, 170 countries were included for the drinking water analysis and 169 countries for the sanitation analysis, equivalent to 99.5% and 98.4% of the global population respectively (Table 2). Countries with populations under 100,000 and/or under 100 km^2^ total area [13] were excluded from the analysis and all countries without any data for water and/or sanitation coverage were excluded.

**Table 2:** Number and description of countries included and excluded from water and sanitation analyses.

Sanitation	
Countries included in raw JMP data (2011)	224
Countries excluded by size or population criteria	49
Countries excluded for missing all data	6
Countries used to calculate cluster averages of shared to improved sanitation ratio	146
Countries missing shared sanitation estimates	37
Countries gap-filled using clustering methodology	25
Countries gap-filled using MDG region and HDI methodology	12
Countries missing data for certain years and technologies	41
Total countries used in analysis	169
Total world population included in analysis	98.4%

Water	
Countries included in raw JMP data (2006)	229
Countries excluded by size or population criteria	56
Countries excluded for missing all data	2
Countries used to calculate cluster averages of proportion of protected wells on plot	22
Countries gap-filled using clustering methodology	148
Countries gap-filled using MDG region and HDI methodology	22
Countries missing data for certain years and technologies	16
Total countries used in analysis	170
Total world population included in analysis	99.5%

### Description of data

The JMP assembles country-level data from national censuses and nationally representative household surveys collected by national offices of statistics and international survey initiatives [4]. The JMP sanitation data were downloaded directly from the JMP website (www.wssinfo.org). Although data for urban and rural improved drinking water, and for water piped to home/plot are publicly available, this data alone is insufficient for the purposes of estimating household versus community-level access for water and sanitation as done here. The additional and more detailed data required for this analysis (including urban and rural population using protected wells, public standpipes, protected springs and rainwater collection) were obtained directly from the JMP for 1990 through 2006; these are the only years for which JMP has produced these more detailed data on drinking water access.

### Statistical Analysis

Where certain country specific data was unavailable, we allocated the mean value derived from a cluster of comparable countries. Countries were allocated to five ‘WatSan’ clusters' according to a previously published methodology based on similarity across a set of WatSan indicators using a hierarchical clustering method and gap statistic analysis [14]. This approach to clustering is reported as being more compact and better separated than comparable geographic or income-based clustering approaches as used by the United Nations and the World Bank [14]. 22 countries included in this analysis were not included in the WatSan clusters developed by Onda and colleagues [14] and were instead grouped by Human Development Index (HDI) category and MDG region (Table 3) to identify mean cluster-level values for missing values. These country clusters were used to estimate mean cluster-level values for the proportion of the population using shared sanitation and household-level protected wells, as described further below.

**Table 3:** Allocation of 151 countries to WatSan clusters for gap-filling.

Cluster	Countries
1	Australia, *Austria*, *Belgium*, Canada, Cyprus, *Czech Republic*, *Denmark*, *Estonia*, Finland, *France*, *Germany*, Greece, *Iceland*, Ireland, Israel, *Japan*, *Latvia*, Lithuania, *Luxembourg*, *Malta*, *Netherlands*, New Zealand, *Norway*, Portugal, Singapore, *Slovak Republic*, Slovenia, South Korea, *Spain*, *Sweden*, *Switzerland*, *United Kingdom*, United States
2	Argentina, *Belarus*, ***Brazil***, Bulgaria, Chile, *Colombia*, ***Cuba***, Iran, ***Kazakhstan***, *Mexico*, Oman, *Russia*, *Ukraine*, *Uruguay*, Venezuela
3	*Albania*, Algeria, *Armenia*, ***Azerbaijan***, *Bosnia and Herzegovina*, *Costa Rica*, *Croatia*, *Dominican Republic*, ***Egypt***, *El Salvador*, FYR Macedonia, *Georgia*, ***Iraq***, *Jordan*, *Kyrgyzstan*, Lebanon, Maldives, *Mauritius*, *Moldova*, *Mongolia*, *Sri Lanka*, ***Syria***, ***Tajikistan***, *Tunisia*, *Turkey*, Turkmenistan, Uzbekistan, *Vietnam*
4	Belize, *Bhutan*, *Bolivia*, *Botswana*, *China*, *Ecuador*, *Gabon*, *Guatemala*, ***Guyana***, ***Honduras***, ***India***, *Indonesia*, *Jamaica*, *Namibia*, *Nicaragua*, *Panama*, *Paraguay*, *Peru*, *Philippines*, *Sao Tome and Principe*, *South Africa*, ***Suriname***, ***Thailand***, *Trinidad and Tobago*
5	Afghanistan, Angola, ***Bangladesh***, *Benin*, *Burkina Faso*, *Burundi*, ***Cambodia***, Cape Verde, *Central African Republic*, *Chad*, *Comoros*, *Congo*, *Cote d'Ivoire*, *Democratic Republic of the Congo*, *Djibouti*, Equatorial Guinea, *Ethiopia*, *Gambia*, ***Ghana***, *Guinea*, *Guinea-Bissau*, ***Haiti***, *Kenya*, *Laos*, *Lesotho*, *Liberia*, *Madagascar*, *Malawi*, *Mali*, *Mauritania*, *Morocco*, *Mozambique*, *Myanmar*, ***Nepal***, *Niger*, *Nigeria*, *Pakistan*, Papua New Guinea, ***Rwanda***, *Senegal*, *Sierra Leone*, *Sudan*, *Swaziland*, Tanzania, ***Timor*** **-** ***Leste***, *Togo*, *Uganda*, ***Yemen***, *Zambia*, *Zimbabwe*

Countries in italics were used to establish cluster averages for shared sanitation; countries in bold were used to establish cluster averages for shared protected wells. The italicized countries are those for which the percentage using shared sanitation were available from JMP. The bolded countries are those for which the percentage of protected wells shared between households could be determined from DHS and MICS survey data.

Where sanitation values were not provided by the JMP for certain countries in certain years (most often 1995, 2000 and/or 2010) estimates were made using simple linear regression techniques based on available data points with Microsoft Excel (2011) [15]. In the 37 countries where there is no data for shared sanitation, the JMP reports these countries as having no shared sanitation [16]. In this analysis, we instead applied a mean cluster value for the ratio of community-level improved sanitation to all improved sanitation (Table 4, Equation 1).

**Table 4:** Equations used for analysis.

Estimate		Equation
1.	Ratio of community-level ‘improved’ sanitation to all ‘improved’ sanitation	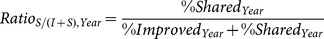
2.	Baseline estimates for 1990 for community level (Scenario 1) sanitation	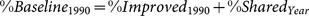
3.	Baseline estimates for 1990 for household level (Scenario 2) water	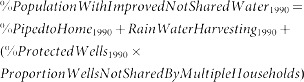
4.	Target coverage estimates for 2015	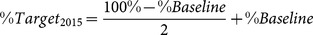

Water values for missing years were estimated using linear regression as described above for sanitation. However, the publicly available JMP estimates for water distinguish only between urban and rural ‘improved’ drinking water access, and between piped water at home/on-plot and all other ‘improved’ categories (protected wells, public standpipes, protected springs and rainwater collection). Estimates of the country-level fraction of ‘protected wells’ that are shared between households are not available in any publication or data set; therefore, raw data from country-level surveys were used. Raw data from Demographic and Health Surveys (DHS) [17] (questions 101 and 103) and Multiple Indicators Cluster Surveys (MICS) [18] (questions WS1 and WS3) from 22 countries were used. Results from these countries were used and gap-filling was conducted for both scenarios as described in Table 3. For the developed countries of WatSan cluster 1, it was assumed that all protected wells were at the household-level (Table 3).

**Figure 1:**
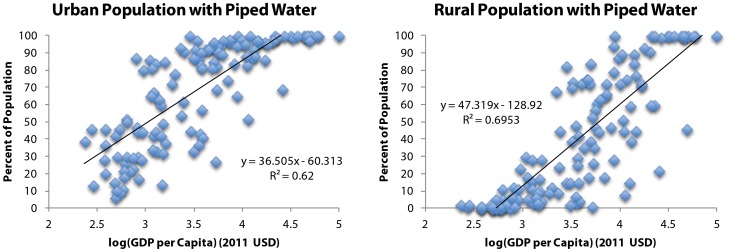
Regressions of urban and rural % piped water coverage vs. GDP per capita.

For some countries urban and rural ‘improved’ water estimates were provided by JMP but data for piped water and other ‘improved’ categories were not. The relationships between country-level urban piped water and log_10_ GDP per capita and rural piped water and log_10_ GDP per capita are strongly positively correlated; therefore, the global regression of these parameters was used to estimate urban and rural piped water coverage when country-level estimates were unavailable from JMP (Fig. 1); this method was used for four countries (Table 5). For these four and another 24 countries that lacked JMP estimates for the other categories of improved water, the distribution of the fraction of ‘other improved’ into the four remaining categories (protected well, public standpost, protected spring and rainwater) was calculated for each MDG region. The corresponding MDG regional averages were then applied to allocate ‘other improved’ to the four remaining categories (Table 6).

**Table 5:** Countries where “% Piped” was estimated from regression equations (see Fig. 1) and the resulting estimates.

Country	MDG region	Log (GDP)	Urban % Improved	Rural % Improved	Estimate Urban % Piped	Estimate Rural % Piped	Final Urban % Piped	Final Rural % Piped
**Australia**	Developed Country	4.78	100	100	114.29	97.39	100	97
**Kuwait**	Western Asia	4.80	99	99	114.79	95.09	99	95
**Qatar**	Western Asia	4.97	100	100	120.98	102.78	100	100
**Libya**	Northern Africa	4.00	54	55	85.64	58.77	54	55

The maximum country-level “% Piped” value was set to the “% Improved” value reported by JMP.

**Table 6:** Mean proportion breakdown of “Other Improved” drinking water access into four categories (public standpost, protected well, protected spring and rainwater collection) in urban and rural areas of each MDG region.

MDG Region	Urban fraction public standpost	Urban fraction protected well	Urban fraction protected spring	Urban fraction rainwater collection	Rural fraction public standpost	Rural fraction protected well	Rural fraction protected spring	Rural fraction rainwater collection
Dev. Countries	0.423	0.365	0.180	0.033	0.194	0.563	0.206	0.037
Eurasia	0.486	0.433	0.072	0.009	0.309	0.612	0.079	0
LAC	0.417	0.496	0.011	0.076	0.229	0.570	0.074	0.127
Oceania	0	0.063	0.063	0.938	0	0.174	0.522	0.304
S.S. Africa	0.671	0.266	0.059	0.004	0.307	0.525	0.139	0.030
W. Asia	0.246	0.505	0.089	0.16	0.409	0.386	0.050	0.155
Northern Africa	0.194	0.122	0.050	0.0629	0.207	0.273	0.054	0.037

Countries (n = 28) for which values were calculated using the mean proportions: Australia, Canada, Switzerland, Germany, Finland, United Kingdom, Ireland, Japan, Lithuania, Luxembourg, Romania, Kazakhstan, Bahamas, Belize, Guadeloupe, French Guiana, Fiji, French Polynesia, Solomon Islands, Vanuatu, Samoa, Equatorial Guinea, Israel, Kuwait, Lebanon, Qatar, Libya, Poland.

With assigned values for all missing data, progress under Scenario 1 (with a community-level benchmark for both water and sanitation) and Scenario 2 (with a household-level benchmark for both water and sanitation) were estimated in accordance with JMP methods [2], [19]. For Scenario 1, we combined estimates for ‘improved’ and ‘shared’ sanitation (Table 4, Equation 2) and compared coverage with that under the standard community-level water benchmark used for the MDG target. For Scenario 2, water access at the household level was calculated by summing JMP estimates for ‘piped to home/plot’ with those for ‘rain water harvesting’ and the estimated fraction of ‘protected wells’ not shared by multiple households (Table 4, Equation 3). Gap-filling was conducted for both scenarios using data from the countries identified in Table 3.

For both scenarios, values for the MDG baseline year (1990) were calculated in accordance with JMP methods for both benchmarks. Based on this revised baseline, values for the MDG target year (2015) were calculated for both scenarios in accordance with the JMP methodology and the same formula was applied to both (Table 4, Equation 4). Estimates of global coverage under the two scenarios were likewise calculated using the linear regression methods used by JMP.

## Results

We estimated global progress between 1990–2015 on community and household-level access to water and sanitation, further disaggregated by rural and urban areas. Using these alternative benchmarks, we were also able to estimate whether the MDG target, refashioned in accordance with these alternative benchmarks, would be met under these two scenarios. Finally, we estimated when universal access to water and sanitation would be achieved using these two benchmarks.

**Figure 2:**
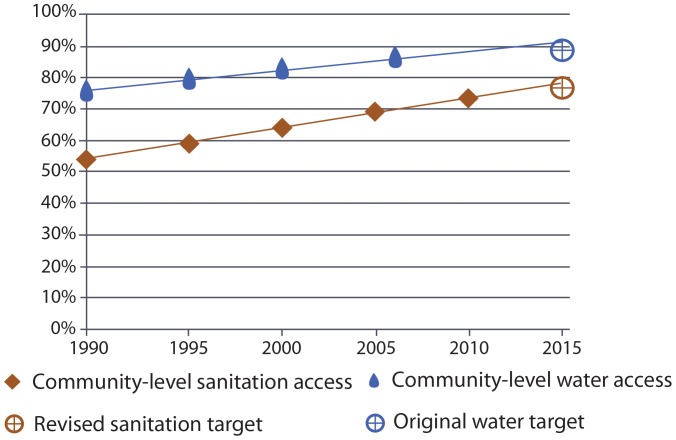
Global progress with a community-level benchmark for water and sanitation (Scenario 1).

Global progress on community-level drinking water and sanitation (see Table 1 for definitions) is depicted in Fig. 2. Under this scenario, with equivalent community-level benchmarks for both water and sanitation, the nominal MDG target of halving the proportion without access is met by 2010 for water and in 2014 for sanitation; both ahead of the 2015 target date. While the water target is met earlier, the rate of progress for sanitation exceeds that of water such that the difference in the proportion with access to water versus sanitation is almost halved between 1990 and 2015. Under this scenario, we estimate that in 2015 the global population without access to water and sanitation at the community-level will be approximately 517 million and 1.58 billion, respectively. We estimate that at current rates of progress, universal community-level access will not be achieved until 2025 and 2037 for water and sanitation respectively.

**Figure 3:**
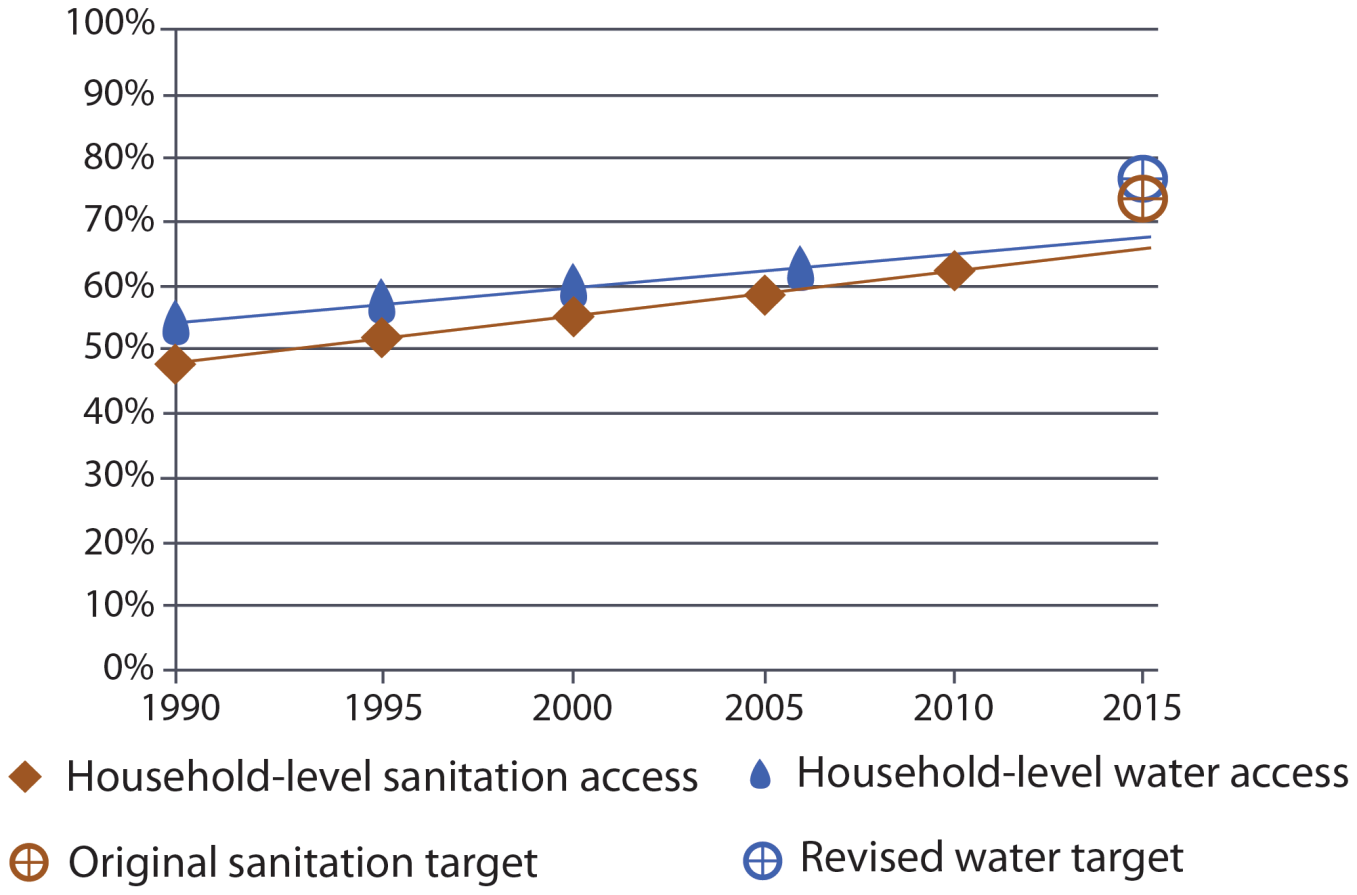
Global progress with a household-level benchmark for water and sanitation (Scenario 2).

An equivalent household-level access benchmark for both water and sanitation was considered under Scenario 2 (see Table 1 for definitions). Global progress against this benchmark between 1990 and 2015 is shown in Fig. 3. Under this scenario the estimated levels of access in 2015 are almost equal such that approximately one third of the world's population will lack access to safe water and sanitation at a household level, equivalent to 2.35 billion and 2.46 billion for water and sanitation respectively. The rate of progress for household-level access is greater for sanitation compared to water such that, on current trends, access to sanitation at a household-level will overtake that of water in 2022. Based on the same linear regression, we estimate that universal household-level access would not be achieved until 2075 and 2061 for water and sanitation, respectively, should progress continue at the current rate.

**Figure 4:**
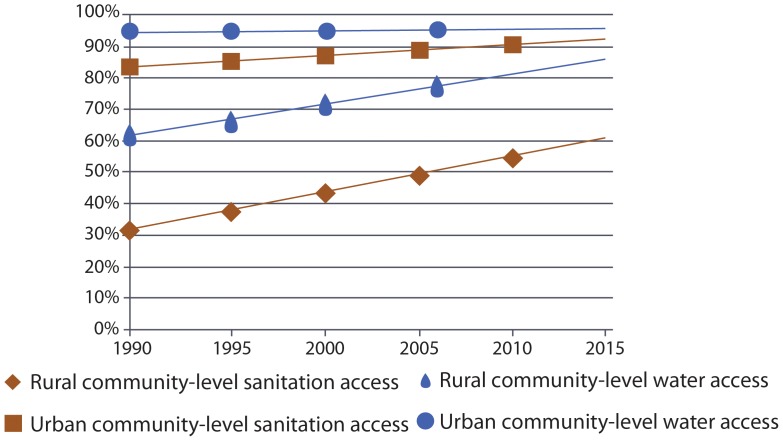
Global progress with a community-level benchmark in rural and urban areas (Scenario 1 & 2).

Globally, levels of access to water and sanitation and rates of progress, whether at a community- or household-level, vary between rural and urban settings. Fig. 4 shows global progress against a community-level benchmark disaggregated by rural and urban settings. In rural areas, between 1990 and 2015 substantial progress has been made on both water and sanitation against a community-level benchmark and the gap between rural and urban levels has reduced substantially. In urban areas, there has been little change in the proportion without community-level access to water whilst the proportion without community-level access sanitation has reduced by half.

**Figure 5:**
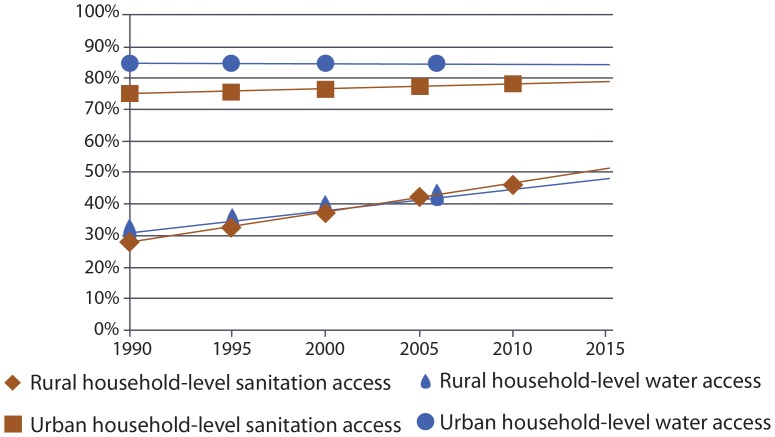
Global progress with a household-level benchmark in rural and urban areas (Scenario 1 & 2).

Between 1990 and 2015, levels of household-level access to water and sanitation improve at an almost equal rate in rural areas (4). Access to rural household-level coverage for water and sanitation rose from approximately 949 million for water and 855 million for sanitation in 1990, to 1.69 billion to 1.80 billion respectively in 2015. Although the proportion of the population in urban areas gaining household-level access to both water and sanitation has changed little for sanitation and remained constant for water between 1990 and 2015 (Fig. 5), in absolute numbers, we estimate a large change. Between 1990 and 2015, we estimate that an additional 1.26 billion people will have achieved household-level access to water and 1.29 billion to household-level sanitation in urban areas. These dramatic increases in absolute numbers have barely kept pace with increases in the global urban population. In 2015, we estimate that for water and sanitation respectively 32% and 34% of the global urban population will be without household-level access.

## Discussion

Applying equivalent benchmarks to both water and sanitation reveals remarkable similarity in progress on water and sanitation since 1990 (Figs. 2 & 3). Although the proportion of those with community-level access to water will exceed that of sanitation in 2015, the proportions with household-level access to water and sanitation will be almost equal (Fig. 3). Disparities in levels of access between rural and urban areas are also diminishing (Figs. 4 & 5).

The purpose of this analysis was to assess progress on water and sanitation between the MDG baseline and end date (1990–2015) against different benchmarks of access. The two scenarios constitute alternative benchmarks for monitoring MDG progress, with equivalent levels of access for water and sanitation in each scenario; Scenario 1 to halve the proportion without community-level access by 2015, and Scenario 2 to halve the proportion without household-level access by 2015. If the sanitation benchmark for the MDG target had, like water, considered community-level access, the target would have been met before 2015. If, alternatively, the water benchmark for the MDG target had been household level access, like that for sanitation, the target would be seriously off-track.

The often reported ‘sanitation deficit’ is apparent only when household-level sanitation access is contrasted with community-level water access, as is done under the current MDG target monitoring framework. Our analysis suggests substantial and comparable progress has been made since 1990 under the MDG target on *both* water and sanitation and at *both* levels of community and household access; and, that progress towards MDG attainment is broadly similar under each scenario. Indeed, since 1990, the rate of progress has been greater for sanitation than for water for both community- and household-level access and in both rural and urban areas.

These benchmarks correspond to important differences in the likely benefits associated with water and sanitation. The importance of distance to water source in determining the level of benefits enjoyed by the individual or household has long been recognized. In their seminal study of domestic water use in East Africa, White and colleagues observed that, “diarrhoeal diseases also seem to diminish when water supplies are made more accessible” [11]. This observation was confirmed by the findings of a later review on the effect of water supply that found the *only* water supply interventions to improve health were those where water was made available at or near the home [20]. More recent systematic reviews have found that increased distance to water source was significantly associated with an increased risk of diarrhoeal disease although the reasons for this association could not be elucidated [21], [22]. These findings are supported by an analysis of household survey data from 26 African countries which found that time spent walking to a household's water source was a significant determinant of under-five child health after adjustment for various potentially confounding variables [23]. One of the more persuasive arguments for this apparent jump in health benefits is that it is driven by a dramatic increase in consumption when water is available at the household-level which enables improved domestic hygiene [24].

For sanitation too there is evidence that the benefits of household level access outweigh those offered by community-level access. In a recent systematic review, Heijnen and colleagues found that that individuals using shared sanitation facilities had a higher risk of diarrhoeal disease infection in comparison to those using household facilities [25]. This is supported by common sense about relative ease of access, especially at night and for certain sub-populations (including women, children, people with disabilities and those with chronic diseases). In general, shared facilities are assumed to be less acceptable to populations and therefore less likely to be used, particularly by women [26]. Joshi and colleagues cite two examples of different types of community-level sanitation facility, one in Kenya and one in Bangladesh; the prohibitive costs of the former driving households to unsafe alternatives, and the latter, with inadequate provision for maintenance, presenting an environmental risk to the community [27]. A study of shared toilets in Bhopal, India, including facilities managed by the community, the municipal authority and private providers, found that the ratio of male to female users was 2∶1 and this was consistent for both adults and children [28]. One reason why women, in particular, may opt to not use shared facilities is the associated risk of violence they may experience, whether this be psychological, physical or sexual [29].

Current evidence suggests that household-level access is required to maximize the benefits associated with the use of water and sanitation. Indeed, many benefits are limited or negated by community-level access such that the human rights obligations of governments to progressively realize sufficient, safe, and accessible water and sanitation services will be difficult to ensure without universal household access. The rates of progress for community- and household-level access have been remarkably consistent for water and sanitation, likewise across rural and urban areas. It seems though that underlying assumptions regarding the attainability of household-level access to water have led to a substantially lower level of ambition with regard to water access as compared to sanitation.

Current proposals for the ‘post-2015’ development agenda appear to perpetuate differentiated benchmarks for water and sanitation with regard to community and household-level access [2]. Whilst universal household-level access to sanitation is deemed attainable within the horizons of a post-2015 goal – albeit with a modification to include facilities shared between a small number (<5) of households -, universal household-level water access is not. The linear progress trends estimated here do not confirm the implicit assumption that household-level access to safe drinking water is significantly less attainable than sanitation. Our analysis instead suggests that the same target year for achieving household-level access to water and sanitation is both appropriate and attainable.

### Limitations

Household surveys, such as the DHS or the MICS, are very important source of data with regard to water and sanitation, permitting estimates of progress that are comparable across countries. There are though limitations to datasets that have been assembled from different surveys that have changed incrementally over time [30]. Gunther and Fink in their analysis of the DHS and MICS surveys for 172 countries report the challenges of managing hundreds of different codes for both water and sanitation, and the JMP uses many other household surveys in addition [19]. Lastly, the survey data compiled by the JMP does not have publicly available margins of error such that it was not possible to estimate confidence intervals for our estimates.

Although our methods are largely consistent with those of the JMP, we did adopt alternative methods to address missing values for particular years and/or categories of access and there are limitations to our gap-filling approach. Firstly, as described above, a number of countries were excluded from this analysis due to their size or the fact that no estimates exist for water and sanitation coverage (Table 2). However, less than 1% of the global population was excluded from our water analysis and less than 2% for sanitation, and as only global trends have been reported, any effect of excluding countries on our results will be minimal. Secondly, we clustered most countries based on comparability across a number of water and sanitation characteristics, rather than HDI as is commonly used, in order to allocate values for missing data. Although we excluded a small number of countries (n = 22), our method compares favourably to that used by the JMP with regard to the compactness of clusters [14] and the overall estimate for global progress is likely to be more robust.

The limitations to the data and methods used for this analysis are broadly similar to those facing the JMP and, as the primary purpose of this analysis is to provide comparable estimates of progress using different benchmarks, the findings are not necessarily weakened. Further analysis is needed to accurately assess trends at regional and national levels that may differ substantially from the global patterns discussed here. Whilst this analysis considered only urban:rural disparities, there are other disparities that warrant investigation. Important among these, based on recent analysis by the JMP and others [1], [31]–[33], are socio-economic disparities as captured by a comparison of the levels and rates of progress between wealth quintiles.

## Conclusions

The claim that sanitation lags behind water is largely an artifact of the benchmarks adopted for monitoring progress towards the MDG target. Global progress towards universal access to both safe water and sanitation at the household level, where the health and other benefits are maximized, is inadequate and the deficit is as great for water as it is for sanitation. Aligning future goals, targets and monitoring efforts with this challenge is critical to secure the full health and other benefits offered by water and sanitation. Future benchmarks should be clear and concise; equally meaningful for those households currently without these services and those with responsibility to progressively realize these goals. Expressing the targets for a future global water and sanitation target in equivalent terms of community or household-level access for water and sanitation, rather than the existing terminologies of ‘basic’ or ‘intermediate’, would focus attention on these two critical service thresholds. Rendering future targets equivalent will also reinforce the interdependency of these services, facilitating greater coordination in planning, resourcing and delivery. One path to achieving this may be a global target with a single benchmark set at the critical level of household access and for both water and sanitation together.
